# A prospective study of the impact of serial troponin measurements on the diagnosis of myocardial infarction and hospital and six-month mortality in patients admitted to ICU with non-cardiac diagnoses

**DOI:** 10.1186/cc13818

**Published:** 2014-04-04

**Authors:** Marlies Ostermann, Jessica Lo, Michael Toolan, Emma Tuddenham, Barnaby Sanderson, Katie Lei, John Smith, Anna Griffiths, Ian Webb, James Coutts, John Chambers, Paul Collinson, Janet Peacock, David Bennett, David Treacher

**Affiliations:** 1 Department of Critical Care, King’s College London, Guy’s & St Thomas’ Foundation Hospital, London, UK; 2 King’s College London, Division of Health and Social Care Research, London, UK; 3 NIHR Biomedical Research Centre at Guy’s and St Thomas’ NHS Foundation Trust and King’s College London, London, UK; 4 Croydon University Hospital, Surrey, UK; 5 Department of Cardiology, Guy’s & St Thomas’ Foundation Hospital, London, UK; 6 St George’s University Hospital, Clinical Blood Sciences, London, UK

## Abstract

**Introduction:**

Troponin T (cTnT) elevation is common in patients in the Intensive Care Unit (ICU) and associated with morbidity and mortality. Our aim was to determine the epidemiology of raised cTnT levels and contemporaneous electrocardiogram (ECG) changes suggesting myocardial infarction (MI) in ICU patients admitted for non-cardiac reasons.

**Methods:**

cTnT and ECGs were recorded daily during week 1 and on alternate days during week 2 until discharge from ICU or death. ECGs were interpreted independently for the presence of ischaemic changes. Patients were classified into four groups: (i) definite MI (cTnT ≥15 ng/L and contemporaneous changes of MI on ECG), (ii) possible MI (cTnT ≥15 ng/L and contemporaneous ischaemic changes on ECG), (iii) troponin rise alone (cTnT ≥15 ng/L), or (iv) normal. Medical notes were screened independently by two ICU clinicians for evidence that the clinical teams had considered a cardiac event.

**Results:**

Data from 144 patients were analysed (42% female; mean age 61.9 (SD 16.9)). A total of 121 patients (84%) had at least one cTnT level ≥15 ng/L. A total of 20 patients (14%) had a definite MI, 27% had a possible MI, 43% had a cTNT rise without contemporaneous ECG changes, and 16% had no cTNT rise. ICU, hospital and 180-day mortality was significantly higher in patients with a definite or possible MI.

Only 20% of definite MIs were recognised by the clinical team. There was no significant difference in mortality between recognised and non-recognised events.

At the time of cTNT rise, 100 patients (70%) were septic and 58% were on vasopressors. Patients who were septic when cTNT was elevated had an ICU mortality of 28% compared to 9% in patients without sepsis. ICU mortality of patients who were on vasopressors at the time of cTnT elevation was 37% compared to 1.7% in patients not on vasopressors.

**Conclusions:**

The majority of critically ill patients (84%) had a cTnT rise and 41% met criteria for a possible or definite MI of whom only 20% were recognised clinically. Mortality up to 180 days was higher in patients with a cTnT rise.

## Introduction

Cardiac troponin (cTn) is a sensitive and specific marker of myocardial injury and is firmly established in the diagnosis of myocardial infarction (MI) [1]. Troponin elevation is common in patients in the intensive care unit (ICU) (12 to 85%) and associated with increased morbidity, mortality and length of stay [2-7]. There are multiple potential aetiologies of troponin rises, including cardiac causes, such as acute coronary syndromes (ACS), MI, heart failure and pericarditis/myocarditis, and non-cardiac causes, such as sepsis, pulmonary disease, renal impairment and intracranial events, but MI and sepsis are the most important causes in this patient cohort [8,9].

There is evidence that the mechanism of troponin release and the prognostic significance of an elevated troponin level vary depending on the underlying cause. In patients with severe sepsis, troponin release can occur in the absence of flow-limiting coronary artery disease and may be due to transient loss in membrane integrity with subsequent troponin leakage or microvascular thrombotic injury [4,10,11]. Ver Elst *et al*. performed post-mortem examinations in patients with septic shock and found similar non-specific changes in troponin positive and troponin negative patients, including elongated myocardial fibers and interstitial oedema but no evidence of MI [12]. Other studies not restricted to sepsis showed that 36 to 71% of patients with elevated troponin levels had ischaemic changes on electrocardiogram (ECG) [2,6,13,14], which therefore defines them as MIs by the consensus criteria [1].

In clinical practice, the diagnosis of MI in ICU patients is complicated by the frequent absence of clinical symptoms, the presence of confounding comorbidities and the difficulty of interpreting ECG changes and troponin elevation in the context of critical illness. Two studies by Lim *et al*., conducted at a single centre, found that MI, as defined by raised troponin levels and contemporaneous ischaemic ECG changes, occurred in 26 to 36% of ICU patients [2,14]. Hospital mortality in patients with MI was 43%, compared to 27% in those with elevated troponin only. More than half of the MIs diagnosed by prospective screening were missed by the clinical team, although the associated mortality was similar irrespective of whether the events were recognised or not (39% vs. 35% ICU mortality and 50% vs. 35% hospital mortality, respectively, with non-significant *P*-values).

Our objectives were i) to repeat the study by Lim and colleagues in consecutive patients admitted for non-cardiac reasons to a mixed medical-surgical ICU in the UK, ii) to determine the epidemiology and outcomes of recognised and unrecognised myocardial events as diagnosed by expert cardiologists, and iii) to investigate the association between troponin elevation and contemporaneous sepsis and vasopressor use.

## Material and methods

### Patients

We conducted a prospective observational study in the 30-bed, level 3 multi-disciplinary adult ICU at the St Thomas’ site of Guy’s & St Thomas’ Hospital, a large tertiary referral centre in London, UK. The ICU is a closed ICU led by full time Intensive Care consultants. During a seven-month period between June and December 2010, consecutive adult patients (age ≥18 years) were recruited. Patients with a high probability of cardiac injury or a primary cardiac diagnosis at ICU admission were excluded, specifically those with a clinical diagnosis of MI or out-of-hospital cardiac arrest, patients who were post-cardiac surgery or cardiac intervention and patients admitted following thoracic trauma with a high likelihood of myocardial injury. Patients were also excluded if they had been transferred from an external ICU following >24 hours stay, had previously been admitted to the ICU during their current hospital stay or were expected to remain in ICU for <48 hours.

### Sample collection

During the first week, we measured troponin T and routine blood tests and performed an ECG on a daily basis. During the second week, troponin T and ECGs were taken on alternate days until discharge from ICU, death or for up to two weeks from admission, whichever occurred first. Serial ECGs taken for research purposes were stored in a secure locker in the research office. Neither the troponin results nor the ECGs were available to the clinical team caring for the patient. However, the clinical team was allowed to perform ECGs and troponin measurements separately as clinically indicated.

### Data collection

At enrolment, baseline demographic data (age, gender, ethnicity), known cardiovascular risk factors (ischaemic heart disease (IHD), diabetes, hypertension, any other type of vascular disease), Acute Physiology and Chronic Health Evaluation (APACHE) II score, and admission diagnosis were recorded.

We recorded use of vasopressors and the presence of sepsis (defined as the presence of ≥2 criteria of systemic inflammatory response syndrome (SIRS) and confirmed or suspected diagnosis of infection) at baseline and daily throughout the study period.

### Laboratory analyses

Blood samples for troponin analysis were stored at -70°C until batch analysis in the biochemistry laboratory at St George’s University Hospital, London. Troponin T was measured using the Roche electrochemiluminescent high sensitivity sandwich immunoassay on the Elecsys 2010 (Roche Diagnostics, Indianapolis, IN, USA). The quoted analytical range is 0.003 to 10 μg/L, total coefficients of variations are 1.5 to 3.4% (measured between 0.024 to 2.665 μg/L) and reference range is less than 15 ng/L (99^th^ percentile) [15]. Other laboratory tests were undertaken at Guy’s & St Thomas’ Hospital.

### Interpretation of troponin levels and diagnosis of myocardial events

Troponin T levels were interpreted as raised if ≥15 ng/L, corresponding to the 99^th^ population percentile [1]. Serial ECGs were analysed independently by two senior cardiologists (JCo, IW) at study completion and evaluated for the presence of ischaemic changes consistent with an acute MI using pre-defined criteria. In case of discrepancy, adjudication was undertaken by a third senior cardiologist (JCh). All cardiologists were blinded to the troponin results and clinical details of the patients.

The diagnosis of an acute myocardial event was based on the presence of elevated troponin T ≥15 ng/L and contemporaneous ischaemic ECG changes according to the European Society of Cardiology/American College of Cardiology Committee criteria [1]. Patients were classified into four groups: (i) definite MI, with troponin T ≥15 ng/L and contemporaneous ECG changes consistent with MI, (ii) possible MI, with troponin T ≥15 ng/L and contemporaneous ischaemic changes on ECG but not fulfilling criteria for definite MI, (iii) troponin T rise alone, and (iv) normal, with troponin T <15 ng/L and not falling into the other three groups.

Medical notes were retrospectively reviewed independently by two ICU clinicians (DT, MO) for evidence that the clinical team had considered the possibility of an acute cardiac event based on ECG and/or troponin criteria performed for clinical reasons and/or explicit entries in the medical notes. Based on these results, we distinguished between clinically recognised and unrecognised acute myocardial events.

### Outcomes

All patients were followed up for 180 days. The main outcomes were mortality at discharge from ICU and hospital and at 180 days, and length of stay in ICU and hospital. In addition, we compared the outcomes of patients with clinically recognised and unrecognised cardiac events.

### Ethics

The study was approved by the Research Ethics Committee (REC) at St Thomas’ Hospital. If a patient had the capacity, written informed consent was obtained from the patient prior to enrolment. If a patient did not have the capacity to consent as a result of the underlying critical illness or sedating medication, the opinion of a personal consultee was sought in accordance with Section 32 of the Mental Capacity Act 2005 (UK). In all cases, a personal consultee was someone with a close personal relationship whom the person who lacked capacity would trust with important decisions about their welfare, for example, a spouse or partner, adult child, parent or a close friend. The personal consultee was asked to consider whether the person who lacked capacity would be content to take part or whether doing so might upset them, and to give their opinion on what the past and present wishes and feelings the person who lacked capacity would have been about taking part in the study. In cases where a personal consultee was consulted, patients were asked to give informed consent retrospectively once they regained capacity. If retrospective consent was declined, all collected samples and ECGs were discarded. The REC waived the need for any additional consent in case retrospective consent could not be obtained due to death or lack of capacity. The REC felt it appropriate that these patients were included in the analysis.

### Statistics

Mean and standard deviation are reported for continuous data. Median and inter-quartile range (IQR) are reported for skewed data such as length of stay. Binary data are reported as frequency and percentage values. Pairwise comparisons were made among three groups: MI (combining the definite MI and possible MI groups), elevated troponin T only, and normal (that is, no troponin elevation). For baseline data, the t-test or the Mann-Whitney U test were used as appropriate to analyse continuous data; Pearson’s Chi-square or Fisher’s Exact Test were used as appropriate in case of categorical variables. The Bonferroni method was used where there was multiple significance testing: a *P*-value of less than 0.016 (0.05/*n*, where *n* is the number of tests) was considered statistically significant. Multivariable regression was used to examine the relationships among raised troponin, MI and patient outcome after adjusting for imbalances in baseline factors. Principal component analysis was used to reduce the number of covariates because the sample is small. Similarly, multivariable regression was used to examine the difference on patient outcome between clinically recognised and unrecognised cardiac events. All analyses were done using Stata v.12 (StataCorp, College Station, Texas, USA).

## Results

### Patient population

Over a seven-month period, 165 eligible patients were identified and approached. In 16 cases, the patient or personal consultee declined consent or assent. The remaining 149 patients were enrolled in the study; however, 5 of these patients were excluded from the final analysis. Reasons for exclusion were the presence of ACS on admission to ICU which had not been recognised by the research team (n = 3) and transfer to a specialist ICU in another hospital for advanced liver and neurosurgical care within 48 hours of admission (n = 2).

The mean age of the remaining 144 patients was 61.9 (SD = 16.9); 42.4% were female (Table 1). The mean APACHE II score at admission was 19.4 (SD = 6.2). Acute renal replacement therapy was initiated in 48 patients (32%). The remaining 101 patients had a median serum creatinine 74 μmol/L (range 22 to 345).

**Table 1:** Demographics and baseline data

	**Total (n = 144)**	**Definite MI (n = 20)**	**Possible MI (n = 39)**	**Elevated cTnT only (n = 62)**	**Normal (No cTnT elevation) (n = 23)**	***P*-value**
Age, mean (SD)	61.9 (16.9)	62.3 (14.1)	67.6 (15.6)	64.3 (15.2)	45.4 (16.6)	MI (definite or possible) vs elevated cTnT; <0.001
						MI (definite or possible) vs normal: <0.001
						Elevated cTnT vs normal: <0.001
Female, n (%)	61 (42%)	8 (40%)	16 (42%)	30 (48%)	7 (30.4%)	MI (definite or possible) vs elevated cTnT; 0.39
						MI (definite or possible) vs normal: 0.39
						Elevated cTnT vs normal: 0.14
White ethnicity, n (%)	123 (85.4%)	17 (85%)	34 (87%)	54 (87%)	18 (78.3%)	MI (definite or possible) vs elevated cTnT; 0.92
						MI (definite or possible) vs normal: 0.36
						Elevated cTnT vs normal: 0.32
APACHE II score, mean (SD)	19.4 (6.23)	18.8 (5.47)	20.5 (5.84)	21.2 (5.84)	13.2 (4.60)	MI (definite or possible) vs elevated cTnT; 0.20
						MI (definite or possible) vs normal: <0.001
						Elevated cTnT vs normal: <0.001
Past medical history						
Ischaemic heart disease, n (%)	24 (16.7%)	5 (25%)	8 (21%)	10 (16%)	1 (4.35%)	MI (definite or possible) vs elevated cTnT; 0.41
						MI (definite or possible) vs normal: 0.099
						Elevated cTnT vs normal: 0.28
Hypertension, n (%)	52 (36.1%)	8 (40%)	23 (59%)	19 (31%)	2 (9%)	MI (definite or possible) vs elevated cTnT; 0.014
						MI (definite or possible) vs normal: <0.001
						Elevated cTnT vs normal: 0.048
Diabetes, n (%)	41 (28.5%)	8 (40%)	14 (36%)	18 (29%)	1 (4%)	MI (definite or possible) vs elevated cTnT; 0.34
						MI (definite or possible) vs normal: 0.002
						Elevated cTnT vs normal: 0.018
Any type of vascular disease, n (%)	29 (20.1%)	4 (20%)	12 (32%)	12 (19%)	1 (4%)	MI (definite or possible) vs elevated cTnT; 0.31
						MI (definite or possible) vs normal: 0.031
						Elevated cTnT vs normal: 0.17

APACHE, Acute Physiology and Chronic Health Evaluation; cTnT , cardiac troponin T; MI, myocardial infarction; SD, standard deviation.

### Prevalence of troponin rise

The majority of patients (121 patients (84%)) had at least one elevated troponin T result (cTnT ≥15 ng/L). Of these, 20 (14%) had contemporaneous ECG changes consistent with a definite MI, 39 (27%) had ECG changes consistent with a possible MI and 62 (43%) had elevated troponin levels without contemporaneous ischaemic ECG changes. The mean, median and maximum recorded troponin levels are shown in Table 2.

**Table 2:** Summary of troponin T levels in all patients over the whole study period

		**Troponin T levels (ng/L)**			
**Group**	**N**	Mean (SD)*	Median*	**Minimum of all observations across all days**	**Maximum of all observations across all days**
Definite MI	20	214 (306)	73	9	3,093
Possible MI	39	229 (416)	63	5	3,335
Elevated troponin T only	62	97 (138)	55	0	1,337
Normal	23	3.3 (3.5)	2.4	0	14
ALL	144	133 (271)	45	0	3,335

MI, myocardial infarction; SD, standard deviation.

*Mean and median are of the patients’ mean troponin levels across follow-up period in each group. (Note that this summary of data serves to illustrate the patients’ average troponin levels across follow-up period and are not used in the analyses of patient outcomes).

Of the 121 patients with at least one troponin T measurement ≥15 ng/L, 66 (46%) were in the range 15 to 100 ng/L, while 55 patients (38%) had at least one measurement >100 ng/L, 24 patients (20%) had at least one normal troponin measurement during study follow-up and only 7 (5.8%) had a normal troponin on admission to ICU. None of the patients with definite MI had a normal troponin on ICU admission.

Patients with raised troponin levels were older (mean age 65.8 versus 45.4, *P* <0.001) and had higher severity of illness scores on admission to ICU (mean APACHE II score 20.6 versus 13.2, *P* <0.001) than those without. Hypertension, vascular disease and diabetes were significantly more common in patients with elevated troponin levels compared to those without a troponin rise but there was no significant difference in the prevalence of IHD (Table 1).

### Outcomes

Patients who had an elevated troponin on admission to ICU had a significantly higher ICU and hospital mortality compared to those without (27% versus 3.3%, *P* = 0.003, and 36% versus 3.3%, *P* <0.001, respectively). Patients with a possible or definite MI during their stay in ICU had a higher ICU, hospital and 180-day mortality compared to those with elevated troponin levels without contemporaneous ischaemic ECG changes; however, the differences were statistically not significant (Table 3).

**Table 3:** Outcome according to MI category

	**Total**	**MI (definite or possible MI)**	**Elevated cTnT only**	**No cTnT rise (normal)**	**unadjusted *P*-value**		adjusted *P*-value*
	**(n = 144)**	**(n = 59)**	**(n = 62)**	**(n = 23)**			
ICU mortality, n (%)	32 (22%)	17 (29%)	14 (23%)	1 (4.35%)	MI vs elevated cTnT;	0.43	0.32
					MI vs normal:	0.04	0.16
					Elevated cTnT vs normal	0.081	0.30
Hospital mortality, n (%)	42 (29%)	22 (37%)	19 (31%)	1 (4%)	MI vs elevated cTnT	0.44	0.39
					MI vs normal	0.015	0.070
					Elevated cTnT vs normal	0.032	0.14
180 days mortality, n (%)	51 (35%)	27 (46%)	23 (37%)	1 (4%)	MI vs elevated cTnT	0.33	0.28
					MI vs normal	0.006	0.058
					Elevated cTnT vs normal	0.015	0.13
ICU length of stay, median (IQR)	7 (3 to 12)	8 (3 to 18)	6 (3 to 11)	4 (2 to 10)	MI vs elevated cTnT	0.13	0.13
					MI vs normal	0.07	0.058
					Elevated cTnT vs normal**	0.48	0.36
Hospital length of stay, median (IQR)	22 (10 to 46)	30 (12 to 51)	19.5 (9 to 46)	18 (5 to 38)	MI vs elevated cTnT	0.27	0.42
					MI vs normal	0.17	0.16
					Elevated cTnT vs normal**	0.57	0.37

cTnT, cardiac troponin T; ICU, intensive care unit; IQR, inter-quartile range; MI, myocardial infarction.

*adjusted for age, APACHE II score, hypertension, diabetes and any type of vascular disease (reduced to two principal components in the regression models).

**estimates based on log-transformed outcomes.

There was no statistically significant difference in length of stay in ICU and hospital between the different troponin cohorts (Table 3).

### Comparison between clinically recognised and unrecognised myocardial events

Only 12 (20%) of the 59 study-identified definite and possible MIs were suspected by the clinical team caring for the patient (Table 4). There was no significant difference in mortality between clinically recognised and unrecognised MIs, although the number of clinically recognised events was small. Length of ICU and hospital stay were longer in those with a recognised MI compared to patients who had an MI which remained unrecognised (median of 17 versus 7 days for ICU stay, and 51 versus 18 days for hospital stay); the differences were still significant after adjustment for baseline risk factors (*P* = 0.024 and *P* = 0.030, respectively).

**Table 4:** Comparison between cardiac events recognised by clinical teams and unrecognised events

	**Recognised MI (definite or possible MI) (n = 12)**	**Not recognised MI (definite or possible MI) (n = 47)**	unadjusted *P*-value*	adjusted *P*-value**
ICU mortality, n (%)	2 (17%)	15 (32%)	0.31	0.22
Hospital mortality, n (%)	3 (25%)	19 (40%)	0.33	0.24
180 days mortality, n (%)	5 (42%)	22 (47%)	0.75	0.52
ICU length of stay, median (IQR)	14.5 (8.5 to 20)	7 (3 to 14)	0.030***	0.024***
Hospital length of stay, median (IQR)	48.5 (35.5 to 63.5)	24 (11 to 42)	0.038***	0.030***

ICU, intensive care unit; IQR, inter-quartile range; MI, myocardial infarction.

*P-value from unadjusted regression model where group was recognised and not recognised.

**P-value from adjusted regression model where group was recognised and not recognised; adjusted variables were age, APACHE II score, hypertension, diabetes and any type of vascular disease (reduced to two principal components in regression models).

***estimates based on log transformed outcome.

Of the 12 patients with a clinically recognised MI, all had their troponin measured by the clinical team. Treatment for an ACS was started in three patients, echocardiography was performed in three, two were referred to cardiology for coronary angiography and one patient was started on a glyceryl trinitrate infusion.

### Association with sepsis and vasopressor use

On the first day in ICU, 105 patients (73%) satisfied ≥2 criteria for SIRS. During their stay in ICU, 104 (72%) patients were septic. The diagnosis of sepsis was associated with increased ICU mortality (28% versus 8%) and increased hospital mortality (35% versus 15%).

Treatment with one or more inotropic or vasoactive drug was necessary in 86 patients of whom 98% received norepinephrine and 6 patients were on two or more medications. Use of vasoactive drugs was associated with both ICU and hospital mortality (37% versus 2%, and 44% versus 7%, respectively).

On the day of troponin rise, 100 patients (69%) had sepsis. Their ICU mortality was 28% compared to 9% in patients who were not septic when they had an elevated troponin level. On the day of troponin elevation, 84 patients (58%) were receiving vasopressors. Their ICU mortality was 37% compared to 2% in patients who were not on vasopressors.

Among patients with a possible or definite MI, 68% of patients were septic and 63% were on vasopressors on the day when the criteria for MI were fulfilled, with similar proportions in those with a definite and possible MI. Only 30% of patients who never had a troponin rise received vasopressor treatment at some stage during their stay in ICU.

## Discussion

This study confirms that raised troponin levels are very common among critically ill patients admitted to ICU for non-cardiac reasons and are associated with an increased mortality up to 180 days. The large majority of ICU patients (84%) had one or more elevated troponin values and 41% met the criteria for MI. Only 20% of definite MIs were recognised by the clinical team.

Previous studies have reported a wide range in prevalence of troponin elevation in critically patients. A meta-analysis by Lim *et al*. showed that in 20 studies, elevated troponin was found in a median of 43% (IQR 21% to 59%) of 3,278 patients [3]. Prevalences of 51 to 62% were reported in three studies [7,14,15]. However, these studies used a variety of different assays, thresholds to define elevation and measurement frequencies, and patient groups varied, making direct comparison impossible. Both Reynolds *et al*. and Audimooolam *et al*. used a troponin I assay with a cut-off at or near the 99th percentile whereas Lim *et al*. used troponin T at a cut-off higher than the 99^th^ percentile [7,14,16]. It is possible that troponin T is more frequently elevated at the 99^th^ percentile than troponin I in critically ill patients, potentially due to renal dysfunction [17].

The exact prevalence of real myocardial infarction in critically ill patients is also not known. Lim and colleagues found that 25.8% of all patients in a single mixed medical-surgery ICU had an MI when assessed by clinically-indicated investigations but prevalence was higher at 36% when using protocol-driven screening investigations [2,14]. A study of 26 septic surgical ICU patients reported a 26.9% prevalence of ischaemic ECG and troponin elevation and a further 7.7% with indeterminate ECG changes [18]. Our study in selected patients who were admitted for non-cardiac reasons revealed a 41% prevalence of possible and definite MIs.

One notable finding of our study, which mirrors that of Lim *et al*. and others [14,19], is that only 20% of study-diagnosed MIs were recognised by the clinical team and mortality was no different between recognised and unrecognised events. Diagnosing an MI in critically ill patients is not straightforward. There are no specific guidelines on the interpretation of ECG changes during critical illness and the reliability of ECG interpretation in critically ill patients for the detection of myocardial ischemia is uncertain. Similarly, there are no separate guidelines for patients with renal impairment. The most recent expert consensus guideline for the Universal Definition of Myocardial Infarction acknowledges that “it is often a challenge for the clinician, caring for a critically ill patient with severe single organ or multi-organ pathology, to decide on a plan of action when the patient has elevated cardiac troponin values” [1].

Relatively little research has focused on the patterns of ECG changes and their interpretation. Lim *et al*. established that there was only modest agreement between different operators interpreting the same ECG when they were not provided with other contextual information (troponin measurements, clinical history) [20]. Mehta *et al*. performed a study in 121 patients with sepsis and asked two independent assessors to analyse the ECGs without knowledge of troponin levels [21]. Inter-rater agreement for ischaemia was fair but improved when the troponin results were revealed to them. To our best knowledge, there are no published studies correlating infarct-characterising ECG changes in ICU patients with angiography-proven coronary artery disease. The problem is confounded further by the fact that critically ill patients can display classic ST elevation in the absence of a troponin elevation, for instance, in the context of intracranial haemorrhage [22].

Ammann *et al*. demonstrated, with echocardiography or autopsy as appropriate, that 70% of patients with troponin elevation did not have significant coronary artery disease [23]. However, documented IHD (that is, previously known and recorded at admission) is common in ICU patients in general, as it was in our study group [24].

Several previous papers confirmed that troponin elevation is associated with increased ICU and hospital mortality in critically ill patients [3,5,23,25-29]. Our data demonstrate that the increased risk of dying persists even after discharge from hospital up to at least 180 days with no statistically significant difference between possible and definite MIs. Several potential mechanisms might explain troponin release in critically ill patients. These include demand ischaemia with or without a degree of coronary vasospasm, the effects of catecholamines and sepsis on the myocardium causing membrane leak and microscopic circulatory thrombosis or maldistribution. We noted that patients with MI had higher median and maximum troponin levels than those with troponin elevations alone without contemporaneous ECG changes. Mortality was low in patients who had normal troponin measurements throughout their ICU admission. We also found that significantly more patients with a possible or definite MI were receiving vaso-active drugs at the time of elevated troponin levels compared to patients who did not have a troponin rise. These findings support the hypothesis that a proportion of patients with a troponin rise may indeed have suffered from critical coronary artery disease and the acute illness, sepsis and/or treatment with catecholamines may have acted as a “stress test” and identified a cohort of patients with a very high risk of dying in six months.

The strengths of this study include the selection of patients who were admitted for non-cardiac reasons and the ECG interpretation by two senior cardiologists with adjudication by a third cardiologist in case of disagreement. Patients were also followed for 180 days which is longer than in previous studies.

It is important to acknowledge some limitations, too. Firstly, we conducted a single centre study. Although we enrolled more patients than the majority of previous studies, we were limited in our subgroup analyses by patient numbers. Our recording of smoking history was incomplete, and we did not collect any information on the use of cardio-protective medication. Finally, we were unable to perform Kaplan-Meier analyses and do not know the exact causes of death.

### Clinical implications and future work

In view of the important association between troponin elevation and mortality in patients admitted to ICU without a primary cardiac diagnosis, and the high number of myocardial events which were not recognised by the clinical team, more work is necessary to improve the understanding of the aetiology of troponin increases. It is also necessary to explore whether there is a role for screening and targeted interventions. Although cardio-protective drugs and invasive strategies (coronary angiography and percutaneous intervention) have a clear role in the patient with ACS outside of the ICU, their role in critically ill patients with a raised troponin is still unclear. Many ICU clinicians do not routinely perform either troponin measurement or regular ECGs in patients without a cardiac diagnosis on admission. Our results show that daily troponin measurement in conjunction with regular ECGs may identify patients at high short- and longer-term risk in whom medical treatment according to an ACS protocol and appropriate diagnostic work-up may be beneficial.

## Conclusions

Myocardial infarction is common amongst critically ill patients, is poorly diagnosed and associated with significant short- and long-term mortality. Further research is warranted to characterise better the underlying aetiology of the troponin rise in critically ill patients and to assess the impact of routine screening and targeted interventions.

## Key messages

•Myocardial infarction is common in critically ill patients but poorly recognized in clinical practice.

•Myocardial infarction during critical illness is associated with a significantly increased risk of dying in up to 180 days.

•More work is necessary to evaluate the benefit of routine screening for myocardial infarction and the benefit of targeted interventions.

## Abbreviations

ACS: Acute coronary syndrome; APACHE: Acute Physiology and Chronic Health Evaluation; cTn: Cardiac troponin; cTnT: Troponin T; ECG: Electrocardiogram; ICU: Intensive care unit; IQR: Inter-quartile range; IHD: Ischaemic heart disease; MI: Myocardial infarction; REC: Research ethics committee; SD: Standard deviation; SIRS: Systemic inflammatory response syndrome.

## Competing interests

The authors declare that they have no competing interests.

## Authors’ contributions

DT, DB and MO designed the protocol and led the research project. KL, JS and BS recruited patients and collected the necessary specimens and ECGs. PC and ET performed the laboratory analyses. JCo, JCh and IW analysed the ECGs. DT and MO retrospectively reviewed the medical notes. JP and JL performed the statistical analyses. AG and MT helped with the data interpretation. MT wrote the first draft. All authors helped to draft the manuscript. All authors apart from DB read and approved the final manuscript. DB died before the final manuscript was submitted.
